# Patient-Reported Experience Measures in Adult Inpatient Settings: A Systematic Review

**DOI:** 10.1155/jonm/5166392

**Published:** 2024-11-23

**Authors:** Yichen Kang, Tingyu Guan, Xiao Chen, Yuxia Zhang

**Affiliations:** Department of Nursing, Zhongshan Hospital Fudan University Postal Code: 200032, Shanghai, China

**Keywords:** instruments, measures, patient-centered, patient experience, systematic review

## Abstract

**Background:** Patient-centered ideas have become the key indicator of medical service quality, and patient-reported experience measures are ways to measure how well this idea is being implemented. There are currently numerous adult inpatient experience instruments available, and it is necessary to conduct such systematic reviews to discover any new instruments and help policymakers and researchers increase the likelihood of hearing true patients' voices through appropriate selection of these instruments.

**Objective:** To identify existing adult inpatient experience measures and to critically appraise their development design and psychometric testing results.

**Methods:** EMBASE, PUBMED, Cochrane, CINAHL (EBSCOhost), PsycINFO, and ProQuest were searched from inception to March 2023. A comprehensive review following the Preferred Reporting Items for Systematic Reviews and Meta-Analyses (PRISMA) guidelines was conducted. Studies were identified via specific search terms and inclusion criteria. The methodological quality assessment was evaluated according to the COnsensus-based Standards for the selection of health Measurement INstruments (COSMIN) checklist.

**Results:** A total of 29 articles reporting on 23 instruments were included. Each instrument demonstrated both satisfaction and disappointment during the development process and psychometric testing with the recommended criteria of the COSMIN checklist. Pilot tests and cognitive interviews were ignored or not reported in 9 studies. Only 5 studies evaluated the content validity. Among all measurement properties, internal consistency and structural validity were the two most frequently measured attributes. None of the 29 included studies assessed the responsiveness or measurement error of the scales.

**Conclusion:** Among a variety of adult inpatient experience instruments, only a limited number of studies were methodologically sound. Further research still needs to be conducted for the development and validation of patient-reported experience measures. New quality assessments, such as instrument utility, also should be implemented to provide a more complete evaluation of instruments in the information era.

## 1. Rationale

The concept of patient-centered care is widely recognized as the foundation of a high-quality healthcare system [1, 2]. Patient experience serves as a key indicator of both medical service quality [3–5] and effective implementation of patient-centered philosophy [6]. Patients, as participants and recipients of healthcare services, can provide abundant feedback which could give direct suggestions to improve the quality of care [7, 8]. Therefore, the concept of patient experience is increasingly being adopted by healthcare stakeholders worldwide to gain valuable and constructive insights [9].

In many kinds of literature, the patient experience is defined as “*the sum of all interactions, shaped by an organization's culture, that influence patient perceptions across the continuum of care*” [10–13]. It is a continuous interactive behavior collective between patients and various healthcare services that is closely linked to patients' expectations and needs [14]. A positive patient experience is strongly associated with increased adherence to treatment plans, fewer complaints against healthcare institutions, and improved patient outcomes [15]. Therefore, in-depth exploration of patient experience can help healthcare providers identify patients' real needs and make targeted improvements, fostering positive patient experiences and ultimately creating a virtuous circle [16]. In contrast to methods for obtaining limited patient experience data such as qualitative studies [5, 17], quantitative tools such as scales and questionnaires can collect and analyze large amounts of patient information in a more standardized way and help healthcare practitioners identify problems more effectively [18, 19]. This is why different patient experience measures, which analyze whether and how often healthcare behaviors occur, have emerged to obtain a more accurate and realistic picture of patients' expectations and needs [20, 21].

Many countries have emphasized the importance of patient experience early and have developed measures to assess the quality of care provided by healthcare professionals. Numerous patient-reported experience measures (PREMs), whether translated or self-developed [22], appeared to improve healthcare quality. PREMs are defined as measures used to record patients' perceptions of their personal experience of the health care they receive [23]. Currently, PREMs are formally leveraged to inform pay-for-performance (P4P) and improve health system performance in many countries [24]. These measures are extensively used across all levels of healthcare performance frameworks, from individual activities to strategic development, highlighting the growing emphasis that policymakers and stakeholders place on patient experience globally [2, 23].

However, the development and testing of PREMs are challenging tasks that require the consideration of many complex psychometric issues [25]. Instruments need to be valid and reliable; otherwise, they cannot consistently provide accurate results or effectively monitor improvements over time [22]. Many scales and questionnaires have been developed and utilized in different countries nowadays, but their quality remains unclear. Especially for older instruments, it may be necessary to update or even discard certain items of the scale. While some systematic reviews have been conducted to assess the psychometric testing and quality of PREMs, many of them select only one specific department of the hospital or one type of patient to evaluate [26–28], leaving a gap in the comprehensive evaluation of PREMs across the entire adult inpatient population. Additionally, some reviews are out of date, and it is necessary to conduct systematic reviews to discover new instruments and articles [22].

The COSMIN checklist is a tool for measuring the methodological quality of studies with standards referring to design requirements and statistical methods [29]. It is also one of the most rigorous and most-commonly tools for evaluating patient-reported outcomes and experience measures in health research [30]. Thus, this review aimed to systematically map PREMs that measure adult inpatient experiences and to assess the quality of the instruments and any bias in the study design and psychometric testings of PREM under the instructions of the COSMIN checklist. This finding will ultimately help policymakers and researchers increase the possibility of the patients' perspective improving hospital quality of care by means of appropriate instrument selection.

### 1.1. Objectives

1. To identify existing instruments for measuring adult inpatient experiences;
2. To identify studies conducted to examine the measurement properties of instruments;
3. To critically appraise quality and bias of each instrument by using the COSMIN checklist.

## 2. Methods

This systematic review was performed in accordance with the Preferred Reporting Items for Systematic Reviews and Meta-Analyses (PRISMA) guidelines [31]. This review was registered with PROSPERO (registration number: CRD42023416310).

### 2.1. Search Strategy

The search was conducted in EMBASE, PubMed, Cochrane, CINAHL (EBSCOhost), PsycINFO, and ProQuest from the inception of each database until March 2023. The search strategy was designed with the assistance of our institution's reference librarian. A combination of subject headings (MeSH subject terms in PUBMED) and keywords were retrieved from different databases. The search construct developed by Terwee, et al. [32] was utilized to guide the search strategy: (1) construct search: patient experience AND (2) population search: inpatients AND (3) instrument AND (4) measurement properties AND (5) exclusion filter. The exclusion filter mainly limited unsuitable publication types, languages, and subject groups. The full search strategies can be found in Supporting Information 1.

### 2.2. Study Selection

All of the references were imported into EndNote (Version X9.3.3, Clarivate Analytics), and duplicates were removed. Two authors (GTY and KYC) independently screened the articles against the eligibility criteria. When the title and abstract were not informative enough to decide, the full-text article was retrieved and reviewed. A third reviewer handled the final decision if necessary. The reference lists of the articles eligible for inclusion in this review were also screened.

The following inclusion criteria were applied:

• Population was hospitalized adult patients.
• Described the development and evaluation of PREMs.
• Published in English.
• Labelled as a satisfaction scale, but framed around measuring patients' experiences.

The following exclusion criteria were applied:

• Review articles, editorials, discussions, opinion papers, and conference proceedings.
• Articles with no full text.
• Instruments developed specifically for elderly individuals or children.
• Instruments developed in specific health facilities (i.e., pediatrics and adolescents, psychiatry, ICU, emergency, outpatient, operating room, obstetrics, etc.).
• Instruments to measure patient satisfaction, expectations, and quality of care.

### 2.3. Data Extraction

Data were extracted separately from the included articles by two authors (GTY and KYC). A standardized data extraction form made in Excel was used to collect all the relevant information from the included articles. The following data were extracted for each instrument: title, author, publication year, country, patient types, response scales and scoring methods, domains, number of items, response rate, mode and timing of administration, and measurement properties. All extracted data were checked for accuracy by a third reviewer. The interrater agreement (Cohen's Kappa) between the two reviewers was 0.81. In accordance with Landis and Koch [33], the threshold for substantial agreement was set at 0.61. Discrepancies in the extracted data were discussed between the two reviewers, or adjudicated by a third reviewer if necessary.

### 2.4. Methodological Quality Assessment

The COSMIN checklist, which aims to evaluate the methodological quality and evaluative applications of health measurement instruments [34, 35], was selected for the assessment of the development, validity, and reliability of the adult inpatient experience instruments in our study.

The COSMIN checklist contains ten boxes that represent 10 measurement properties, including instrument design, content validity, structural validity, internal consistency, cross-cultural validity, reliability, measurement error, criterion validity, hypothesis testing, and responsiveness. A four-point scoring system is used to rate each standard as “very good,” “adequate,” “doubtful,” “inadequate,” or “not applicable” [36]. The overall rating is determined by the lowest rating of any item in the box (“worst score counts”) [36]. Detailed instructions for the use of the COSMIN checklist are available on the COSMIN website (https://www.cosmin.nl/).

Two independent reviewers (GTY and KYC) performed the COSMIN evaluation. The interrater agreement (Cohen's Kappa) among the two reviewers ranged from 0.68 to 0.75. Discrepancies of opinion were resolved by consensus between the two reviewers or, if no consensus was reached, with the help of a third reviewer (CX). The results of this assessment were organized according to the tables provided in the COSMIN user manual (https://www.cosmin.nl/).

## 3. Results

**Figure 1:**
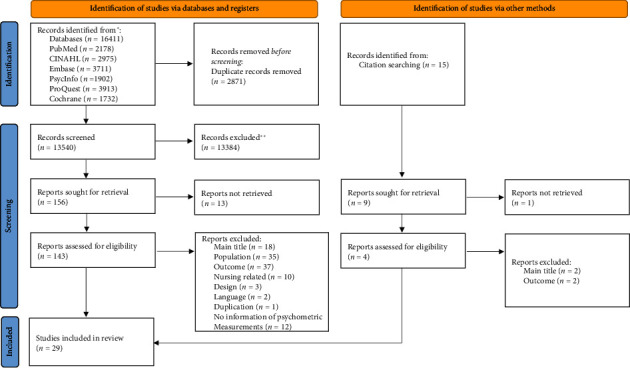
PRISMA flowchart.

A total of 16,411 studies were identified from the database, and 15 studies were identified through citation tracking. After the final selection using the inclusion and exclusion criteria for the review, 29 studies were included. Certain instruments, such as HowRwe, which utilizes the “excellent” scoring approach [37], were excluded due to their scoring methods. Although it was included in another systematic review [38], it does not align with our definition of patient experience and has been excluded from consideration in this manuscript. Please refer to Figure 1 for the PRISMA results.

### 3.1. Characteristics of the Search Result

We included a total of 29 studies involving 23 instruments [1, 2, 4, 6, 7, 14, 16–19, 21, 25, 39–55]. These instruments originated from various regions of the world such as the USA, England, Germany, and Norway. Most of these instruments generated several versions during their development. However, we selectively included studies that provided a more complete evaluation of the relevant psychometric measurements of the established tool. For example, the Picker Patient Experience Questionnaire (PPE-15) has undergone many iterations, so we included three articles to present its more comprehensive development and psychometric validation process.

The number of items ranged from 6 to 127. The Outcomes and Experiences Questionnaire (QEQ-E) [42], Nordic Patient Experiences Questionnaire [48], and the Generic Short Patient Experiences Questionnaire [47] had the fewest items, with only 6, 8, and 10 items, respectively, making them suitable for rapid screening of patient experiences. In contrast, the Experienced Patient-Centeredness Questionnaire (EPAT) [21], developed in 2021, comprises 121 items, including various aspects of the patient experience. All population groups were inpatient populations, among which the Cancer Patient Experiences Questionnaire is specifically developed for the inpatient experiences of cancer patients [4] and the Coronary Heart Disease In-patient Experience Questionnaire [43] is for coronary heart disease inpatients. Mail is the most common mode of administration of these instruments, followed by face-to-face interviews, and telephone surveys. There is also a notable use of email and mixed modes such as online or paper-based options. For example, face-to-face interviews, and online-based or mail interviews are all available at Hospital Consumer Assessment of Healthcare Providers and Systems (HCAHPS). The Hong Kong Inpatient Experience Questionnaire (HKIEQ) [53, 54] also provides telephone assistance and home-based, face-to-face interviews for patients.

The scoring methods for the questionnaires were predominantly “always–never,” followed by “agree–disagree,” with the majority of questionnaires utilizing a mixed scoring approach. With respect to the domains covered in each instrument, “Communication,” “Information,” “Coordination of Care,” “Patient involvement and emotional support,” “Safety and environment,” and “Professional Competence” were the most measured domains. All instruments covered aspects of the technical and interpersonal components of quality of care, and each tool may have its own specific focus on different aspects. For example, the EPAT measures and pays more attention to patient involvement and emotional support, such as the involvement of family and friends, the uniqueness of each patient, the empowerment of patients, support of mental well-being, and collaboration as equal partners and involvement in decision making. For specific details on the basic characteristics of the studies, refer to Tables 1 and 2.

### 3.2. Methodological Quality of the Included Studies

#### 3.2.1. PREM Development and Content Validity

In 18 studies reporting on PREM design, only 9 studies satisfied the standards for a COSMIN rating of “very good” or “adequate,” which means that these articles elaborated their general design and concept elicitation clearly and thoroughly [1, 2, 17, 21, 40–42, 50, 54]. The other 9 studies were conducted under the guidance of a construct such as a theory or a conceptual framework, but qualitative or quantitative research on the relevance and comprehensiveness of the item content in these studies was insufficient [4, 16, 18, 19, 25, 45, 47, 51, 53]. Only 9 studies performed cognitive interview studies and pilot tests to ensure the comprehensibility and comprehensiveness of the PREM [1, 2, 4, 16, 39, 40, 42, 47, 51], and only five articles evaluated the quality of content validity [1, 2, 17, 40, 42]. In the COSMIN checklist, content validity involves asking patients about relevance, comprehensiveness, and comprehensibility and asking professionals about relevance and comprehensiveness. Most of the studies evaluated only the content validity from professionals and neglected the patients.

#### 3.2.2. Internal Consistency

The methods employed to assess the psychometric properties varied among the included studies. Among the eight COSMIN risk-of-bias checklist criteria for good measurement properties, 20 studies met between one and five criteria. Not a single study met all of the COSMIN criteria. Internal consistency and structural validity were the two most frequently measured attributes among all measurement properties. Twenty studies assessed internal consistency, ranging from 0.39 to 0.97, revealing substantial heterogeneity in the internal consistency of all scales. Ten instruments showed sufficient internal consistency as required by the COSMIN checklist (Cronbach's alpha(s) ≥ 0.70 for each unidimensional scale or subscale) [6, 7, 17, 18, 25, 39, 42, 48, 50, 52], such as PPE-15 and Nordic Patient Experiences Questionnaire.

#### 3.2.3. Structural Validity

Seventeen studies evaluated structural validity, with 11 studies conducting exploratory factor analysis and six studies performing confirmatory factor analysis (CFA). Three studies reporting CFA have sufficient structural validity as required by the COSMIN checklist (the CFI or TLI or a comparable measure > 0.95 OR RMSEA < 0.06 OR SRMR < 0.008) [2, 14, 41]. However, no studies used the item response theory (IRT) method to measure structural validity.

#### 3.2.4. Reliability

Reliability was evaluated among 8 researches, ranging from (0.36–0.96). Four instruments reporting reliability have sufficient structural validity as required by the COSMIN checklist [14, 25, 45, 47, 48].

#### 3.2.5. Other Measurement Properties

Thirteen studies measured construct validity [4, 6, 14, 16, 19, 25, 40, 42, 43, 48, 50, 52, 54]. Few articles reported cross-cultural validity/measurement invariance and criterion validity in their psychometric testing, only PEQ was measured cross-cultural validity/measurement invariance [14], and 4 assessed criterion validity [14, 44, 49, 50]. None of the 29 included studies assessed the responsiveness and measurement error of the scales. The results of the measurement properties and quality assessment of each instrument are presented in Tables 3 and 4.

## 4. Discussion

This systematic review provides an overview and a critical analysis of all available instruments for adult inpatient experience. Twenty-nine articles and 23 instruments were identified and subjected to an appraisal of their quality by the means of COSMIN checklist. New instruments such as PREM-CCH [50] and EPAT [21], which were developed separately in China and Germany, and several classic scales, such as PPE-15 [57], HCAHPS [46], and PEQ [18], are all included in this review.

One of the primary challenges the research team encountered during literature selection and screening was the inconsistent definition of patient experience. Several articles either lacked a precise definition or conflated patient experience with related concepts such as patient satisfaction, perception, involvement, and quality of care, although what they truly measured was patient experience. Same situation also occurred in response scales or scoring methods used in different instruments. Generally, response options like “never,” “sometimes,” and “always” align with the concept of patient experience, yet some questionnaires inappropriately use terms such as “agree” to rate the frequency of healthcare issues. Thus, the determination of the concept and exploration of the response scale still require further investigation.

Most people believe that the fewer items a scale contains, the more willing patients are to complete it, naturally leading to higher-quality results [58]. However, after testing different lengths of scales, there was no direct relationship between patient acceptance and the number of items [5]. Additionally, no direct relationship was found between scale length and response rate. For example, the PPE-15, a short version of the original questionnaire, still covers all the basic domains of measuring patient experience and provides decision makers with rich information for health service improvement [18].

Each instrument demonstrated both satisfaction and disappointment during the development process and psychometric testing with the recommended criteria of the COSMIN checklist. Reassuring, all published instruments have conducted and reported psychometric testing and other information such as the utility of the instrument.

In the PREM design part, most design necessities and methods were met. For example, relevant theories, conceptual frameworks, and the use of cognitive interviews or pre-experiments were used to test the initial scales. However, asking patients and professionals about relevance, comprehensiveness, and comprehensibility of the scale are often not evaluated thoroughly, and none of the studies reported a very complete experimental and testing process. This led to lower scores in content validity and scale design part. Content validity is the degree to which the content of an instrument adequately reflects of the construct being measured [35]. It refers to the relevance, comprehensiveness, and comprehensibility of the instrument for the construct, target population, and context of use of interest. Measuring content validity is considered to be the most important issue in the COSMIN criteria [29] because a lack of content validity affects all other measurement properties. What's more, poor content validity may also lead to unacceptable construct validity, reliability, a low response rate, and biased responses [59]. Therefore, it is imperative that the development of PREMs should build on high-quality content validity. Surprisingly, we found that many classic or widely used tools lack sufficient content validity, unlike the instruments developed recently. This is not an uncommon phenomenon [59], and one possible reason is that these questionnaires were developed earlier, when requirements for reliability and validity were less required.

Structural validity, internal consistency, and reliability were found to be the most measured indicators. The majority of studies used exploratory or CFAs (other used item–item, item–dimension, dimension–dimension, and/or item–total correlations) to test for construct validity instead of using IRT methods. IRT is a collection of measurement models that attempt to explain the connection between observed item responses on a scale and an underlying construct [60]. It is a relatively new and unfamiliar theory for most researchers, so very few researchers have attempted to use it for assessment, and more evidence-based research is needed to confirm its applications. However, the measurement of these properties is still insufficient, and the psychometric robustness of an instrument must be based on a thorough assessment of all psychometric properties [27].

Few articles measured cross-cultural validity/measurement invariance and criterion validity. One of the most likely reasons for rarely reporting criterion validity is that there is currently no universal and gold standard for its assessment [61]. Measurement invariance testing is used to evaluate the assumption that scales operate similarly across groups [62]. Fewer instruments were available for this item possibly because we included mostly adult inpatients without specific subgroup population. Disappointingly, none of the studies reported evidence of responsiveness or measurement error. Both indicators are important for instrument validation. Responsiveness refers to the ability of an instrument to detect change over time [28]. For instruments used repeatedly or as a national measurement, testing responsiveness should be prioritized. Measurement error demonstrates that the intervention/program/service was clinically relevant for improving the patient experience. Currently, patient experience is internationally recognized as a key determinant of healthcare quality. The clinical relevance of improving patient experience will have implications for resource allocation for quality improvement. Thus, measurement error should be evaluated especially when patient experience scores are used to inform decision making.

Some articles or instruments showed poor ratings according to the COSMIN criteria. However, this did not mean that they were not recommended at all, because the COSMIN checklist uses the “lowest score counts” principle for an overall score. It is also worth noting that our measurement results rely on patients themselves, but from a patient perspective, the experience or constitution of quality is likely to change over time [22]. Thus, to ensure the validity and reliability of the instrument, re-examination and re-verification are highly suggested.

## 5. Implications for Future Studies

More studies evaluating the responsiveness and measurement error are needed because these two properties were not assessed in any of the scales mapped in this study. IRT or Rasch analysis is also warranted to assess the structural validity according to the COSMIN methodology. Without the information about the nonviolation of assumptions underlying IRT/Rasch analysis, the structural validity of a study cannot be considered sufficient even when the model shows an adequate fit [63]. At present, several electronic patient self-reported experience and outcome systems have been developed, and self-optimization of the scale has been carried out in combination with IRT and Computerized Adaptive Testing (CAT) to flexibly provide personalized scale content for patients and reduce their sense of boredom in answering questions [56]. The integration, extraction, analysis, and other advanced functions of such information systems can better assist in providing directions for clinical nursing decision-making research [64]. However, in the process of combining instruments with electronic information systems, the results obtained may differ from the original regular version, so a large number of experiments are needed for verification and exploration in the later stage [65]. What's more, future research must address how to ensure the consistent use of the instruments developed and how to maintain their validity.

## 6. Implications for Nursing Management

This systematic review will inspire nursing managers to pay more attention to the PREMs. By evaluating the strengths and limitations of existing PREMs, nursing managers can gain deeper insights into the factors that influence patient experience and the effectiveness of interventions aimed at improving it. High-quality PREMs can provide accurate and reliable data that reflect the true concerns and needs of patients, which in turn enhances patient trust and strengthens the patient–provider relationship. Moreover, high-quality PREMs contribute to better decision making by providing nursing managers with robust data for resource allocation, staff training, and policy development. From a policy-making perspective, the findings of this review can inform the development of guidelines and standards that ensure that PREMs are used to their full potential. This includes establishing benchmarks for patient experience that promote transparency, accountability, and continuous quality improvement within healthcare institutions.

## 7. Limitations

Our systematic review has several limitations. In fact, the development and implementation of the instruments will vary depending on the healthcare policies of different countries, hospitals, or departments. Therefore, in addition to the psychometric testing and the assessment of COSMIN checklist, we should also consider some other real-world factors such as the healthcare systems, costs, research environments, and user acceptability [16, 66]. For example, the utility of the instrument should be considered in real-world practice. Van der Vleuten [67] identified five aspects of instrument utility: validity, reliability, cost-efficiency, acceptability, and educational impact. Each of these aspects is important to users of PREMs. Therefore, it may be better for us to evaluate these instruments by using COSMIN checklist as well as other criteria to provide an all-round assessment.

## 8. Conclusion

Patient experience has become increasingly important in national healthcare service assessment. It is urgent for most countries to find accurate and verified instruments for measuring patient experience. This systematic review provided a whole map of current PREMs and their quality assessments to assist stakeholders in selecting the best-suited instrument. Further research on the development and validation of PREMs still needs to be conducted. A combination of assessment methods may be required in future instrument quality assessments, especially in the information age.

## Figures and Tables

**Table 1:** Instrument overview.

Instrument/abbreviation	Author (year)	Country	Patient types	No. of items	Response rate	Mode of administration	Timing of administration
Hospital Consumer Assessment of Healthcare Providers and Systems/HCAHPS	Rothman et al. [46]	USA	Adult inpatient	29	Not reported	Face to face	Discharge day
CQI (Consumer Quality Index) Inpatient Hospital Care Questionnaire/Dutch HCAHPS	Smirnova et al. [6]	Dutch	Adult inpatient	38	31.70%	Online or paper‐based	Hospitalized for at least 24 h with a discharge within the previous 12 months
Picker Patient Experience Questionnaire/PPE-15	Jenkinson, Coulter, and Bruster [18]	UK, USA, Germany, Sweden, Switzerland	Adult inpatient	15	48.00%	Mail	Within 1 month of discharge
	Wolf et al. [52]	Sweden	Adult inpatient		66.00%	Email	One month after discharge
	Bertran et al. [39]	Spanish and Catalan	Adult inpatient		44.00%	Email	Within 1 week discharged from hospital/clinic
Patient Experience Questionnaire/PEQ	Pettersen et al. [16]	Norway	Medical and surgical inpatient	35	53.00%	Mail	Six weeks after discharge
	Addo, Mykletun, and Olsen [14]	Norway	Adult inpatient	33	54.92%	Mail	After discharge
Generic Short Patient Experiences Questionnaire/GS-PEQ	Sjetne [47]	Norway	Adult inpatient	10	43.60%	Mail	After discharge
Patient Experiences Questionnaire for Interdisciplinary Treatment for Substance Dependence/PEQ-ITSD	Haugum et al. [25]	Norway	Inpatient with interdisciplinary treatment for substance dependence	22	91.40%	Mail	On-site
Nordic Patient Experiences Questionnaire/NORPEQ	Oltedal et al. [45]	Norway	Adult inpatient	8	48.80%	Mail	Within a 3-week period of receiving inpatient treatment at the same Norwegian hospital
	Skudal et al. [48]	Finland, Norway, Sweden, and the Faroe Islands			Not reported	Mail	One week before their planned discharge, 2 weeks postdischarge
The Cancer Patient Experiences Questionnaire/CPEQ	Iversen, Holmboe, and BjertnæS [4]	Norway	Cancer inpatients	127	52.00%	Mail	Not reported
In-Patient Experience Questionnaire	Labarère et al. [19]	French	Medical and surgical inpatient	29 (surgical) 28 (medical)	71.40%	Mail	Within 2–4 weeks of discharge
	Labarere et al. [55]	French		30	71.00%	Mail	Within 2–4 weeks of discharge
Hong Kong Inpatient Experience Questionnaire/HKIEQ	Wong et al. [53]	China–Hong Kong	Inpatients	58	28.00%	Telephone—92% (471 patients) Home-based, face-to-face interview—8% (40 patients)	Discharged from hospitals within 48 h to 1 month prior to the interview
	Wong et al. [54]	China–Hong Kong	Inpatients	18	70.20%	Phone	Discharged from hospitals within 48h to 1 month prior to the interview
In-Patient Assessment of Health Care/I-PAHC	Webster et al. [51]	Ethiopian	Inpatient in low-income settings	12	95.00%	Face-to-face interviews	At the time of discharge
NHS Adult In-Patient Survey/NHS-AIPS	Sullivan, et al. [49]	England	Medicine general surgery	76	49.00%	Not reported	Not reported
The Coronary Heart Disease In-patient Experience Questionnaire/I-PEQ (CHD)	Jenkinson et al. [43]	England	Coronary heart disease inpatient	37	74.30%	Mail	After an inpatient episode for CHD
Health Services & InPatient Experience/HS&PE	Colucciaa, et al. [7]	Italy	Adult inpatient	19	Not reported	On-site	On the day of discharge
Patient-Reported Experience Measure for Care in Chinese Hospitals/PREM-CCH	Wang, et al. [50]	China	Inpatients	19	Not reported	Not reported	Not reported
Flemish Patient Survey/FPS	Bruyneel et al. [41]	Belgium	Surgical, medical, maternity, specialty service, geriatrics	27	Not reported	On-site	Not reported
Quality of Care Patient Questionnaire/QoC	Villiers-Tuthill et al. [2]	Turkey, Greek, Portugal, Romania, Croatia, Macedonia, Bulgaria	Inpatients	12 + 1(open)+1(general)	Not reported	Electronic/paper	Not reported
Experienced Patient‐Centeredness Questionnaire/EPAT	Christalle et al. [21]	German	Four groups of chronic diseases: cancer, cardiovascular diseases, mental disorders, or musculoskeletal disorders	125 items (inpatient version has 121 items)	Not reported	Not reported	Not reported
Outcomes and Experiences Questionnaire/OEQ-E	Gibbons et al. [42]	England	Adults	6	32.60%	Not reported	Not reported
Updated Valuing Patients as Individuals Scale/uVPAIS	Jones et al. [1]	England	Inpatients	31 (lengthy) 10 (short)	37%	Mail	On discharge or early return to their home
Quality from the Patient's Perspective Questionnaire/QPP	Wilde and Larsson [44]	Sweden	Inpatients	22	79%	Mail	Before leaving the hospital
Neurorehabilitation Experience Questionnaire for Inpatients/NREQ	Kneebone, et al. [17]	England	18–65 adults	16	Not reported	First: interview Second: mail	One week before their planned discharge, 2 weeks postdischarge
Quality of Trauma Care Patient-Reported Experience Measure/QTAC-PREM	Bobrovitz et al. [40]	Canada	Adult (age ≥ 16 years) patients with a primary diagnosis of injury discharged alive from hospital	31	78%	Paper-based survey	Within 3 days of planned discharge

**Table 2:** Instrument domains and scoring methods.

Instrument/abbreviation	Domain									Response scale and scoring methods
	Communication	Information	Coordination of care	Involvement and emotional support	Safety and environment	Professional competence	Admission and discharge	Pain management and physical comfort	Others	
Hospital Consumer Assessment of Healthcare Providers and Systems/HCAHPS	Communication with nurses; communication with doctors; communication about medicines	Discharge information	/	/	Hospital environment	Hospital staff responsiveness	/	Pain management	Overall rating of hospital; willingness to recommend hospital	Never, sometime, usually, always
CQI (Consumer Quality Index) Inpatient Hospital Care Questionnaire/Dutch HCAHPS	Communication with nurses; communication with doctors; communication about medication	Explanation of treatment	/	Own contribution	Feeling of safety	/	Admission; discharge information	Pain management	/	Dichotomous scale: 1 = yes, 2 = no Four-point Likert scales: 1 = never, 2 = sometimes, 3 = usually, 4 = always
Picker Patient Experience Questionnaire/PPE-15	/	Information and education	Coordination of care	Emotional support; respect for patient preferences; involvement of family and friends	/	/	Continuity and transition	Physical comfort	Overall impression	No, sometime, often, always; 100-point ordinal response (overall impression)
Patient Experience Questionnaire/PEQ	Communication	Medication information; examination information	/	Contact with next-of-kin	Hospital and equipment; organization	Doctor services; nursing services	Future complaints	/	General satisfaction	Not at all (1)–to a very large extent (5)
Generic Short Patient Experiences Questionnaire/GS-PEQ	/	Information	Organization	User involvement	/	Clinician services	Outcome; incorrect treatment	Accessibility	/	
Patient Experiences Questionnaire for Interdisciplinary Treatment for Substance Dependence/PEQ-ITSD	/	/	/	Personnel	Milieu	Treatment	Outcome	/	/	Not at all (1)–to a very large extent (5)
Nordic Patient Experiences Questionnaire/NORPEQ	/	Information on tests	Nursing care	Doctors and nurses interested in problem	/	Doctors' professional skills; nurses' professional skills	Incorrect treatment	/	General satisfaction	Not at all (1)–to a very large extent (5)
The Cancer Patient Experiences Questionnaire/CPEQ	Nurse contact; doctor contact	Information	Organization	Contact with next of kin	Patient safety; hospital standard	/	/	/	/	Not at all (1)–to a very large extent (5)
In-patient Experience Questionnaire	/	Medical information	Coordination	/	Living arrangements; convenience	Nursing care; physician care	Discharge management	/	/	“Strongly agree” (4) to “strongly disagree” (1) and “does not apply”
Hong Kong Inpatient Experience Questionnaire/HKIEQ	/	Information provision	Coordination of care	Care and involvement in decision making; respect and privacy	Environment and facilities; handling patient feedback	Hospital staff	Prompt access	Physical and emotional needs	Overall care of healthcare professionals and quality of care	Yes, always (1); yes, sometimes (2); and no (3)
In-Patient Assessment of Health Care/I-PAHC	Communication with nurses; communication with doctors; medication and symptom communication	/	/	/	Physical environment	/	/	Pain management	/	Never (1) to always (4); overall evaluation of care (scored 0–10)
NHS Adult In-Patient Survey/NHS-AIPS	/	/	/	Involvement	Cleanliness	Nurses; doctors	/	/	/	100-point ordinal response scales
The Coronary Heart Disease In-patient Experience Questionnaire/I-PEQ(CHD)	Communication	Information	Coordination of care	/	Hospital environment	/	Discharge planning	Pain and physical comfort	/	Dichotomous scale: “no problem” or “problem”
Health Services & InPatient Experience/HS&PE	Communication	/	/	Humanization; enablement	/	/	/	/	/	Never = 1 to always = 5
Patient-Reported Experience Measure for Care in Chinese Hospitals/PREM-CCH	Communication	Information	/	/	Efficiency	Professional competence	Medical costs; health outcomes	/	Hospital recommendation	①Very negative = 1, negative = 2, neither negative nor positive = 3, positive = 4, very positive = 5; ②Never = 1, Sometimes = 2, Often = 3; ③Never = 1, Sometimes = 2, Often = 3, Always = 4; ④No = 1, Yes = 2; ⑤No = 1, I did not care/Not sure = 2, Yes = 3
Flemish Patient Survey/FPS	/	Information about condition; information about treatment and procedures	/	Privacy	Dealing with patients and collaboration between healthcare providers; safe care	/	Discharge; preparing for hospital stay	Pain management	/	Disagree (1) to totally agree (4) + not applicable; never (1) to always (4)
Quality of Care Patient Questionnaire/QoC	/	/	/	Doctor–patient interaction	/	/	Medical investigation and treatment	/	Technical and interpersonal aspects of care	Strongly disagree/disagree/neither agree nor disagree/agree/strongly agree
Experienced Patient‐Centeredness Questionnaire/EPAT	Appropriate communication	Personally tailored information	Good planning of care	Consideration of personal circumstances; involvement of family and friends; uniqueness of each patient; empowerment of patients; support of mental well-being; collaboration as equal partners and involvement in decision making; trustful relationship	Integration of additional healthcare elements; patient safety	Teamwork of healthcare providers	Access to care	Support of physical well-being	/	Completely disagree (1) to completely agree (6)
Outcomes and Experiences Questionnaire/OEQ-E	Communication	Information	/	Involvement; responsiveness to individual needs; discussion of worries and fears	/	/	/	/	/	Scores range from 0 to 18 with high scores indicating a good experience
Updated Valuing Patients as Individuals Scale/uVPAIS	/	/	/	Care and respect; understanding and engagement; patient concerns	/	/	/	/	/	Strongly agree (5) to strongly disagree (1)
Quality from the Patient's Perspective Questionnaire/QPP	/	/	/	Identity-oriented approach	Socio-cultural atmosphere	Medical–technical competence	/	Physical–technical conditions	/	Do not agree at all (1) to completely agree (4) of little or no importance (1) to of the very highest importance (4)
Neurorehabilitation Experience Questionnaire for Inpatients/NREQ	/	/	/	Ownership; personal value	Therapeutic atmosphere	/	Holistic approach	/	/	“Mostly agree”, “not sure,” or “mostly disagree” + “not applicable”
Quality of Trauma Care Patient-Reported Experience Measure/QTAC-PREM	Communication	Information	Equality	Interpersonal care	Safety	/	Transfers and patient transport	Pain management, comfort	/	Never, 0; sometimes, 1; usually, 2; always, 3; no, 0; yes, 3

**Table 3:** Measurement properties of each instrument.

Instruments/abbreviation	Year	Measurement property	Result
Hospital Consumer Assessment of Healthcare Providers and Systems/HCAHPS	2008	Internal consistency	Internal consistency for each domain: nurse communication = 0.85; doctor communication = 0.87; nursing services = 0.71; physical environment = 0.49; pain control = 0.81; communication about medicines = 0.73; discharge information = 0.72; coordination of care = 0.70

CQI (Consumer Quality Index) Inpatient Hospital Care Questionnaire/Dutch HCAHPS	2017	Internal consistency	Cronbach's alpha coefficient individual (department) for each domain: Admission = 0.77 (0.81); communication with nurses = 0.83 (0.87); communication with doctors = 0.81 (0.84); own contribution = 0.69 (0.80); explanation of treatment = 0.81(0.89); pain management = 0.79 (0.86); communication about medication = 0.68 (0.85); feeling of safety = 0.64 (0.64); information at discharge = 0.76 (0.82)
		Structural validity	Individual level: CFI = 0.96; TLI = 0.95; RMSEA = 0.04 Department level: CFI = 0.83; TLI = 0.81; RMSEA = 0.06
		Construct validity	Surgery (*n* = 3225) individual level: CFI = 0.98; TLI = 0.98; RMSEA = 0.03 Cardiology (*n* = 2697) individual level: CFI = 0.97; TLI = 0.96; RMSEA = 0.03 Internal medicine (*n* = 1984) individual level: CFI = 0.98; TLI = 0.98; RMSEA = 0.03 Obstetrics and gynecology (*n* = 643) individual level: CFI = 0.98; TLI = 0.97; RMSEA = 0.02

Picker Patient Experience Questionnaire/PPE-15	2002	Internal consistency	UK = 0.86; Switzerland = 0.83; Sweden = 0.80; Germany = 0.85; USA = 0.87
	2012	Internal consistency	Cronbach's alpha coefficient was 0.87 for the overall scale
		Construct validity	Good self-rated health (SRH) and having Swedish as native language were associated with better care experiences and poorer experiences with greater healthcare utilization, higher age, functional impairment, and being female.
	2018	Internal consistency	Cronbach's alpha coefficient of the overall scale was 0.84 (CI^4^ 0.81–0.87); specifically for Catalan version was 0.84 (CI 0.81–0.87); and for Spanish 0.83 (CI 0.79–0.87)
		Structural validity	Four factors extracted which explained 43% of the total variance

Patient Experience Questionnaire/PEQ	2004	Structural validity	Exploratory factor analysis: 20 items represented by six factors and accounted for 67% of the total variance
		Internal consistency	Cronbach's alpha coefficient individual (department) for each domain: information future complaints = 0.82; nursing services = 0.80; communication = 0.74; information examinations = 0.72; contact with next-of-kin = 0.78; doctor services = 0.71; hospital and equipment = 0.78; information medication = 0.67; Organization = 0.61; general satisfaction = 0.83
		Reliability	Intraclass correlation coefficient (ICC) for each domain: information future complaints = 0.62; nursing services = 0.85; Communication = 0.62; information examinations = 0.76; contact with next-of-kin = 0.69; doctor services = 0.72; hospital and equipment = 0.79; information medication = 0.65; organization = 0.74; general satisfaction = 0.63
		Construct validity	Unadjusted scores differed on all summed rating scales for the oldest versus the youngest half of the patients, gender, and fulfillment of expectation. Adjusting scores did not change the results.
	2021	Structural validity	CFI = 0.95; TLI = 0.94; RMSEA = 0.04; PCLOSE^5^ = 1.00 (model 2)
		Cross-cultural validity/measurement invariance	Model 2 produced best fitness values for all indices and it had excellent configural invariance and metric invariance. It did not have scalar invariance.
		Reliability	Composite reliability (CR) for each domain in Model 2: nurse services = 0.90; doctor services = 0.92; information = 0.87; organization = 0.81; next of kin = 0.83; standard = 0.82; discharge = 0.87; interaction = 0.72
		Convergent validity	Average variance explained (AVE) for each domain in Model 2: nurse services = 0.57; doctor services = 0.64; information = 0.70; organization = 0.53; next of kin = 0.70; standard = 0.44; discharge = 0.77; interaction = 0.57
		Discriminant validity	Three dimensions (“doctor services,” “organization,” “standard”) were not distinct from the others enough for each to measure the different subconcepts under patient experience in Model 2.
		Construct validity	Item loadings ranged from 0.88 (on “discharge”) to 0.55 (on “standard”).
		Criterion-related validity	Overall patient experience and each individual dimension related to and predicted at least one outcome variable positively and significantly.

Generic Short Patient Experiences Questionnaire/GS-PEQ	2011	Content validity	Test and modified with inpatients on applicability and comprehensiveness.

Patient Experiences Questionnaire for Interdisciplinary Treatment for Substance Dependence/PEQ-ITSD	2017	Structural validity	Two factors explained 51.8% of the variance and outcome factors explained 73.4% of the variance
		Reliability	Test–retest reliability for each domain: treatment and personnel = 0.85; milieu = 0.84; outcome = 0.82
		Internal consistency	Cronbach's alpha coefficient of each domain: treatment and personnel = 0.91; milieu = 0.75; outcome = 0.91
		Construct validity	The results of the study were generally consistent with the hypothesis. The associations between the scale scores and the tested variables were statistically significant in 17 out of 18 tests.

Nordic Patient Experiences Questionnaire/NORPEQ	2007 and 2012	Structural validity	Principal component analysis and component loadings were used and measured.
		Internal consistency	Cronbach's *α* for the six NORPEQ items ranged from 0.84 to 0.88 for Finland and the Faroe Islands, respectively.
		Reliability	Intraclass correlation for the NORPEQ scores was 0.85.
		Construct validity	It assessed the correlation of NORPEQ scores with patient perceptions of satisfaction, incorrect treatment, expectations, health, and outcomes.
The Cancer Patient Experiences Questionnaire/CPEQ	2012	Structural validity	EFA (exploratory factor analysis): six factors explained 63% of the total variance CFA (confirmatory factor analysis): *x*^2^ = 26697.60, *p* < 0.001, df = 621, RMSEA = 0.083, GFI = 0.81, CFI = 0.97, and IFI^7^ = 0.97
		Internal consistency	Cronbach's alpha coefficient of each domain: nurse contact = 0.93; doctor contact = 0.93; information = 0.94; organization = 0.82; patient safety = 0.67; contact with next of kin = 0.85; hospital standard = 0.74
		Reliability	Test–retest reliability (ICC) for each domain: nurse contact = 0.83; doctor contact = 0.85; information = 0.78; organization = 0.85; patient safety = 0.62; contact with next of kin = 0.81; hospital standard = 0.75
		Construct validity	Statistically significant associations based on explicit hypotheses provided evidence for the construct validity of the scales.

Inpatient Experience Questionnaire	2004	Structural validity	Seven principal components accounted for 62.4 percent of the total variance First component accounted for 37.2 percent of the total variance
		Construct validity	Empirical hypotheses derived from the literature or formulated by the authors were tested.
		Internal consistency	Cronbach's alpha coefficient of each domain: medical information = 0.90; nursing care = 0.86; living arrangements = 0.67; discharge management = 0.73; coordination = 0.62; physician care = 0.73; convenience = 0.39
	2001	Structural validity	Six scales accounted for 58% of the variance in total satisfaction scores.
		Internal consistency	Cronbach's alpha coefficient of each domain: nursing care = 0.84; communication = 0.86; discharge continuity = 0.82; physician care = 0.78; living arrangements = 0.72; convenience = 0.67

Hong Kong Inpatient Experience Questionnaire/HKIEQ	2013	Internal consistency	Cronbach's alpha coefficient (*α*) of the overall scale was 0.75. Cronbach's alpha coefficient (*α*) of each domain: access, choice, coordination = 0.97; communication and information = 0.77; privacy = 0.80; involvement in decisions = 0.72; physical comfort and pain relief = 0.49; environment and facilities = 0.52; involvement of family and friends = 0.52; support for self-care = 0.68; care of healthcare professionals and feedback handling = 0.80
		Reliability	Test–retest reliability of the overall scale was 0.75. Test–retest reliability of each domain: access, choice, coordination = 0.96; communication and information = 0.62; privacy = 0.36; involvement in decisions = 0.90; physical comfort and pain relief = 0.40; environment and facilities = 0.67; involvement of family and friends = 0.49; support for self-care = 0.85; care of healthcare professionals and feedback handling = 0.65
		Structural validity	Nine dimensions of hospital care explaining 75.4% of the variance.
	2014	Internal consistency	KR-20 coefficient of the summative scores across items within the core set was 0.86
		Construct validity	Spearman's rank correlation coefficient for the relationship between the summative scores in the short-form and the HKIEQ: *ρ* = 0.92 (*p* < 0.05)

In-Patient Assessment of Health Care/I-PAHC	2011	Internal consistency	Cronbach's alpha coefficient (*α*) of each domain in I-PAHC: Communication with nurses = 0.85; communication with doctors = 0.86; physical environment = 0.54; pain management = 0.88; medication communication = 0.70
		Convergent validity	Nearly all associations between each of the summary scores and the item assessing patients' overall evaluations of the healthcare experience for I-PAHC scales were statistically significant.

NHS Adult In-Patient Survey/NHS-AIPS	2013	Internal consistency	Cronbach's *α* estimated the correlation of patient-level item scores within a domain and is an indicator of the amount of domain score variance that is due to common factors.
		Criterion validity	Criterion validity of AIPS was retained for most specialty subgroups.

The Coronary Heart Disease In-patient Experience Questionnaire/I-PEQ (CHD)	2002	Internal consistency	KR-20 coefficient of each domain: pain = 0.65; hospital environment = 0.66; information and communication = 0.74; patient involvement = 0.64; coordination = 0.6; discharge and transition = 0.71
		Structural validity	All domains loaded on a single factor explaining 52% of the variance.
		Construct validity	It distinguished between groups as was hypothesized.

Health Services & In-Patient experience/HS&PE	2013	Internal consistency	Cronbach's alpha coefficient of the overall scale was 0.94.
		Structural validity	EFA: 59.6% was explained of the variance CFA: RMSEA = 0.08; CFI = 0.91; TLI = 0.91

Patient-Reported Experience Measure for Care in Chinese Hospitals/PREM-CCH	2021	Internal consistency	Cronbach's alpha coefficient of the whole inpatient PREM-CCH was 0.9. Cronbach's alpha coefficient for each domain: communication and information = 0.8; professional competence = 0.7; medical costs = 0.8; efficiency = 0.8; health outcomes = 0.8
		Structural validity	Exploratory factor analysis was used.
		Construct validity	All individual items in the revised inpatient PREM-CCH had statistically significant correlations with the item on general satisfaction with hospital care
		Criterion validity	Total score for the instrument correlated moderately with the score for the item on general satisfaction with hospital care (rho = 0.63).

Flemish Patient Survey/FPS	2017	Structural validity	EFA&CFA; CFA: CFI = 0.98; TLI = 0.98; RMSEA = 0.044
		Measurement invariance	Different dimensions of patient experiences were conceptualized similarly over time and did not depend on the mode of administration, nursing unit/ward, or any of the included patient characteristics.

Quality of Care Patient Questionnaire/QoC	2017	Structural validity	CFA: CMIN/DF = 2.462; CFI = 0.946; GFI = 0.967; AGFI = 0.947; RMSEA = 0.053; PCLOSE = 0.355
		Content validity	Expert consensus, review of the literature, and patient focus groups from all countries involved through the development process.
		Internal consistency	Cronbach's alpha coefficient: healthcare professional communication and interpersonal care (*α* = 0.69), and physical hospital facilities (*α* = 0.61)
Experienced Patient‐Centeredness Questionnaire/EPAT	2021	Content validity	CVI: 7 items had a CVI of 0.5 or lower; 5 items had a mean feasibility to assess rating below 2; 15 items had a mean relevance rating below 3.

Outcomes and Experiences Questionnaire/OEQ-E	2015	Construct validity	Study 1: Correlations between the OEQ-E and the change score for the disease-specific measures were OHS = 0.23; OKS = 0.29. OEQ-O was slightly correlated with the EQ-5D change scores for each condition (hip 0.16,*p* < 0.05; knee 0.14, *p* < 0.01, both not significant) Study 2: Lowest correlations were observed for OEQ-E with NHS inpatient survey items addressing cleanliness and access and waiting.
		Content validity	Patient focus groups involved through the development process.
		Internal consistency	0.89 for the OEQ-E domain

Updated Valuing Patients as Individuals Scale/uVPAIS	2018	Internal consistency	uVPAIS: care and respect = 0.94; understanding and engagement = 0.92; patient concerns = 0.78 uVPAIS-short: care and respect = 0.90; understanding and engagement = 0.81; patient concerns = 0.69
		Content validity	Focus group involved through the development process.
		Structural validity	Exploratory factor analysis and principal component analysis
		Discriminant validity	Variations in scores between males and females and between White British and other ethnic groups
		Concurrent validity	The six-item general satisfaction subscale of the patient satisfaction questionnaire was used to test concurrent validation examining correlation coefficients with subscales of the uVPAIS questionnaire.

Quality from the Patient's Perspective Questionnaire/QPP	2002	Internal consistency	Cronbach's alpha coefficient: medical–technical competence = 0.67; physical–technical conditions = 0.65; identity-oriented approach = 0.91; socio-cultural atmosphere = 0.72
		Criterion validity	No significant differences were found between the long and short versions.

Neurorehabilitation Experience Questionnaire for Inpatients/NREQ	2012	Internal consistency	Cronbach's *α* was 0.76 at initial testing, and 0.8 at postdischarge
		Reliability	The test–retest reliability between the two time points was 0.78.

Quality of Trauma Care Patient-Reported Experience Measure/QTAC-PREM	2016	Internal consistency	Subscale Cronbach's α ranged from 0.67 to 0.87 for the acute measure subscales and were 0.42 and 0.80 for the post–acute care subscales.
		Reliability	On the acute measure, estimates ranged from 0.42 to 0.88, with 61% of items achieving coefficients greater than 0.65. On the post–acute measure, estimates ranged from 0.55 to 1.00, with 94% of items achieving coefficients greater than 0.65.
		Structural validity	Exploratory factor analysis revealed a three-factor solution accounting for 79% of the variance in the acute care subscale, and a two-factor solution accounting for 83% of the variance in the post–acute subscale.
		Construct validity	Convergent validity with the HCAHPS revealed correlations that were primarily low to moderate, ranging from 0.01 to 0.64.

Abbreviations: AGFI = adjusted goodness-of-fit index, CFI = comparative fit index, CI = confidence interval, CMIN/DF = chi-square minimum/degrees of freedom, CVI = content validity index, GFI = goodness-of-fit index, IFI = incremental fit index, PCLOSE = *p* of close fit, RMSEA = root mean square error of approximation, TLI = Tucker–Lewis index.

**Table 4:** Results of quality assessment.

Instruments/abbreviation	Year	1a	1b	2a	2b	2c	2d	2e	3	4	5	6	7	8	9a	9b	10
Hospital Consumer Assessment of Healthcare Providers and Systems/HCAHPS	2008	N	N	N	N	N	N	N	N	A	N	N	N	N	N	N	N
CQI (Consumer Quality Index) Inpatient Hospital Care Questionnaire/Dutch HCAHPS	2017	N	N	N	N	N	N	N	V	V	N	N	N	N	N	A	N
Picker Patient Experience Questionnaire/PPE-15	2002	N	N	N	N	N	N	N	N	A	N	N	N	N	N	N	N
	2012	N	N	N	N	N	N	N	N	A	N	N	N	N	N	V	N
	2018	N	I	N	N	N	N	N	A	V	N	N	N	N	N	N	N
Patient Experience Questionnaire/PEQ	2004	D	I	D	D	D	D	D	A	V	N	V	N	N	N	I	N
	2021	N	N	N	N	N	N	N	V	N	V	D	N	D	A	V	N
Generic Short Patient Experiences Questionnaire/GS-PEQ	2011	D	I	N	N	N	N	N	N	N	N	N	N	N	N	N	N
Patient Experiences Questionnaire for Interdisciplinary Treatment for Substance Dependence/PEQ-ITSD	2017	D	N	N	N	N	N	N	A	V	N	V	N	N	A	V	N
Nordic Patient Experiences Questionnaire/NORPEQ	2007	D	N	D	D	D	D	N	A	A	N	A	N	N	A	N	N
	2012	N	N	N	N	N	N	N	A	A	N	A	N	N	A	N	N
The Cancer Patient Experiences Questionnaire/CPEQ	2012	D	I	D	D	D	D	D	V	V	N	V	N	N	A	A	N
Inpatient Experience Questionnaire	2004	N	N	N	N	N	N	N	A	V	N	N	N	N	N	V	N
	2001	D	N	D	D	D	D	D	A	V	N	N	N	N	N	N	N
Hong Kong Inpatient Experience Questionnaire/HKIEQ	2013	D	N	D	D	D	N	N	A	V	N	A	N	N	N	N	N
	2014	V	N	N	N	N	A	A	N	A	N	N	N	N	A	N	N
In-Patient Assessment of Health Care/I-PAHC	2011	D	I	D	D	D	D	D	N	V	N	N	N	N	A	N	N
NHS Adult In-Patient Survey/NHS-AIPS	2013	N	N	N	N	N	N	N	N	D	N	N	N	D	N	N	N
The Coronary Heart Disease In-Patient Experience Questionnaire/I-PEQ(CHD)	2002	D	N	N	N	N	N	N	D	V	N	N	N	N	N	D	N
Health Services & InPatient Experience/HS&PE	2013	N	N	N	N	N	N	N	V	A	N	N	N	N	D	N	N
Patient-Reported Experience Measure for Care in Chinese Hospitals/PREM-CCH	2021	A	N	N	N	N	N	N	D	V	N	N	N	A	D	N	N
Flemish Patient Survey/FPS	2017	A	N	N	N	N	N	N	V	N	V	N	N	N	N	N	N
Quality of Care Patient Questionnaire/QoC	2017	A	A	A	A	A	A	A	V	A	N	N	N	D	N	N	N
Experienced Patient‐Centeredness Questionnaire/EPAT	2022	A	N	N	N	N	V	N	N	N	N	N	N	N	N	N	N
Outcomes and Experiences Questionnaire/OEQ-E	2015	A	A	A	A	A	A	A	N	A	N	N	N	N	A	A	N
Updated Valuing Patients as Individuals Scale/uVPAIS	2018	A	A	A	A	A	V	V	A	V	N	N	N	N	A	A	N
Quality from the Patient's Perspective Questionnaire/QPP	2002	N	N	N	N	N	N	N	N	V	N	N	N	D	N	N	N
Neurorehabilitation Experience Questionnaire for Inpatients/NREQ	2012	A	N	A	A	A	A	A	N	V	N	V	N	N	N	N	N
Quality of Trauma Care Patient-Reported Experience Measure/QTAC-PREM	2016	V	V	V	V	V	V	V	A	V	N	V	N	N	V	N	N

*Note:* 1a. PREM design; 1b. Cognitive interview study or other pilot test; 2a. Asking patient about relevance; 2b. Asking patients about comprehensiveness; 2c. Asking patients about comprehensibility; 2d. Asking professionals about relevance; 2e. Asking professionals about comprehensiveness; 3. Structural validity; 4. Internal consistency; 5. Cross-cultural validity\measurement invariance; 6. Reliability; 7. Measurement error; 8. Criterion validity; 9a. Comparison with other outcome measurement instruments (convergent validity); 9b. Comparison between subgroups (discriminative or known-groups validity); 10. Responsiveness. Ratings: V = very good; A = adequate; D = doubtful; I = inadequate; N = not applicable.

## Supporting Information

The supporting material 1 showed the full search strategies of the study which contained search strings and results of each database.

## References

[1] Jones M. C., Williams B., Rattray J. (2018). Extending the Assessment of Patient-Centredness in Health Care: Development of the Updated Valuing Patients as Individuals Scale Using Exploratory Factor Analysis. *Journal of Clinical Nursing*.

[2] Villiers-Tuthill A., Doulougeri K., Mcgee H., Montgomery A., Panagopoulou E., Morgan K. (2017). Development and Validation of a Cross-Country Hospital Patient Quality of Care Assessment Tool in Europe. *Patient*.

[3] Bakshi A. B., Wee S. L., Tay C. (2012). Validation of the Care Transition Measure in Multi-Ethnic South-East Asia in Singapore. *BMC Health Services Research*.

[4] Iversen H. H., Holmboe O., BjertnæS O. A. (2012). The Cancer Patient Experiences Questionnaire (CPEQ): Reliability and Construct Validity Following a National Survey to Assess Hospital Cancer Care from the Patient Perspective. *BMJ Open*.

[5] Jenkinson C., Coulter A., Bruster S., Richards N., Chandola T. (2002). Patients’ Experiences and Satisfaction with Health Care: Results of a Questionnaire Study of Specific Aspects of Care. *Quality and Safety in Health Care*.

[6] Smirnova A., Lombarts K., Arah O. A., van der Vleuten C. P. M. (2017). Closing the Patient Experience Chasm: A Two-Level Validation of the Consumer Quality Index Inpatient Hospital Care. *Health Expectations*.

[7] Coluccia A., Ferretti F., Pozza A., Lorini F. (2013). Health Services & Inpatient Experience (HS&PE): Development of a Patient-Centered Outcome Measure for Inpatient Settings in Italy. *International Journal of Person Centered Medicine*.

[8] Jones C. H., Woods J., Brusco N. K., Sullivan N., Morris M. E. (2021). Implementation of the Australian Hospital Patient Experience Question Set (AHPEQS): a Consumer-Driven Patient Survey. *Australian Health Review*.

[9] Hodgkinson E. L., Boullin P., Heary S., Grant B. (2019). Development and Pilot of a Burns-specific Patient-Reported Experience Measure. *Burns*.

[10] Judan-Ruiz E. A., Mina R. J. L., Macindo J. R. B. (2020). Psychometric Properties of the Filipino Version of Hospital Consumer Assessment of Healthcare Providers and Systems (HCAHPS): A Cross-Cultural Validation Study. *Journal of Patient Experience*.

[11] Oben P. (2020). Understanding the Patient Experience: A Conceptual Framework. *Journal of Patient Experience*.

[12] Wang T., Giunti G., Melles M., Goossens R. (2022). Digital Patient Experience: Umbrella Systematic Review. *Journal of Medical Internet Research*.

[13] Almohaisen N. A., Alsayari N. M., Abid M. H. (2023). Improving Patient Experience by Implementing an Organisational Culture Model. *BMJ Open Quality*.

[14] Addo S. A., Mykletun R. J., Olsen E. (2021). Validation and Adjustment of the Patient Experience Questionnaire (PEQ): A Regional Hospital Study in Norway. *International Journal of Environmental Research and Public Health*.

[15] Wong E. L., Coulter A., Cheung A. W., Yam C. H., Yeoh E. K., Griffiths S. (2013). Item Generation in the Development of an Inpatient Experience Questionnaire: a Qualitative Study. *BMC Health Services Research*.

[16] Pettersen K. I., Veenstra M., Guldvog B., Kolstad A. (2004). The Patient Experiences Questionnaire: Development, Validity and Reliability. *International Journal for Quality in Health Care*.

[17] Kneebone I. I., Hull S. L., Mcgurk R., Cropley M. (2012). Reliability and Validity of the Neurorehabilitation Experience Questionnaire for Inpatients. *Neurorehabilitation and Neural Repair*.

[18] Jenkinson C., Coulter A., Bruster S. (2002). The Picker Patient Experience Questionnaire: Development and Validation Using Data From In-Patient Surveys in Five Countries. *International Journal for Quality in Health Care*.

[19] LabarèRE J., Fourny M., Jean-Phillippe V., Marin-Pache S., Patrice F. (2004). Refinement and Validation of a French In-Patient Experience Questionnaire. *International Journal of Health Care Quality Assurance*.

[20] Aoki T., Yamamoto Y., Nakata T. (2020). Translation, Adaptation and Validation of the Hospital Consumer Assessment of Healthcare Providers and Systems (HCAHPS) for Use in Japan: A Multicentre Cross-Sectional Study. *BMJ Open*.

[21] Christalle E., Zeh S., Hahlweg P. (2022). Development and Content Validity of the Experienced Patient-Centeredness Questionnaire (EPAT)-A Best Practice Example for Generating Patient-Reported Measures From Qualitative Data. *Health Expectations*.

[22] Beattie M., Murphy D. J., Atherton I., Lauder W. (2015). Instruments to Measure Patient Experience of Healthcare Quality in Hospitals: A Systematic Review. *Systematic Reviews*.

[23] Jamieson Gilmore K., Corazza I., Coletta L., Allin S. (2023). The Uses of Patient Reported Experience Measures in Health Systems: A Systematic Narrative Review. *Health Policy*.

[24] OECD (2014). Paying for Performance in Health Care.

[25] Haugum M., Iversen H. H., Bjertnaes O., Lindahl A. K. (2017). Patient Experiences Questionnaire for Interdisciplinary Treatment for Substance Dependence (PEQ-ITSD): Reliability and Validity Following a National Survey in Norway. *BMC Psychiatry*.

[26] Beecher C., Greene R., O’Dwyer L. (2021). Measuring Women’s Experiences of Maternity Care: A Systematic Review of Self-Report Survey Instruments. *Women and Birth*.

[27] Fernandes S., Fond G., Zendjidjian X. Y. (2020). Measuring the Patient Experience of Mental Health Care: A Systematic and Critical Review of Patient-Reported Experience Measures. *Patient Preference and Adherence*.

[28] Male L., Noble A., Atkinson J., Marson T. (2017). Measuring Patient Experience: A Systematic Review to Evaluate Psychometric Properties of Patient Reported Experience Measures (PREMs) for Emergency Care Service Provision. *International Journal for Quality in Health Care*.

[29] Terwee C. B., Prinsen C. A. C., Chiarotto A. (2018). COSMIN Methodology for Evaluating the Content Validity of Patient-Reported Outcome Measures: A Delphi Study. *Quality of Life Research*.

[30] Gram E. G., J Á R., Heiberg Agerbeck A., Martiny F., Bie A. K. L., Brodersen J. B. (2023). Methodological Quality of PROMs in Psychosocial Consequences of Colorectal Cancer Screening: A Systematic Review. *Patient Related Outcome Measures*.

[31] PRISMA (2020). PRISMA 2020 Checklist[EB/OL]. http://prisma-statement.org/documents/PRISMA_2020_checklist.pdf.

[32] Terwee C. B., Jansma E. P., Riphagen I. I., de V. E. T. H. C. (2009). Development of a Methodological PubMed Search Filter for Finding Studies on Measurement Properties of Measurement Instruments. *Quality of Life Research*.

[33] Landis J. R., Koch G. G. (1977). The Measurement of Observer Agreement for Categorical Data. *Biometrics*.

[34] Mokkink L. B., Terwee C. B., Patrick D. L. (2010). The COSMIN Study Reached International Consensus on Taxonomy, Terminology, and Definitions of Measurement Properties for Health-Related Patient-Reported Outcomes. *Journal of Clinical Epidemiology*.

[35] Mokkink L. B., Terwee C. B., Patrick D. L. (2010). The COSMIN Checklist for Assessing the Methodological Quality of Studies on Measurement Properties of Health Status Measurement Instruments: an International Delphi Study. *Quality of Life Research*.

[36] Terwee C. B., Mokkink L. B., Knol D. L., Ostelo R. W., Bouter L. M., de V. E. T. H. C. (2012). Rating the Methodological Quality in Systematic Reviews of Studies on Measurement Properties: A Scoring System for the COSMIN Checklist. *Quality of Life Research*.

[37] Lewis C. G., Inneh I. A., Schutzer S. F., Grady-Benson J. (2014). Evaluation of the First-Generation AAOS Clinical Guidelines on the Prophylaxis of Venous Thromboembolic Events in Patients Undergoing Total Joint Arthroplasty: Experience with 3289 Patients from a Single Institution. *Journal of Bone and Joint Surgery American Volume*.

[38] Bull C., Byrnes J., Hettiarachchi R., Downes M. (2019). A Systematic Review of the Validity and Reliability of Patient-Reported Experience Measures. *Health Services Research*.

[39] Bertran M. J., ViñARáS M., Salamero M. (2018). Spanish and Catalan Translation, Cultural Adaptation and Validation of the Picker Patient Experience Questionnaire-15. *J Healthc Qual Res*.

[40] Bobrovitz N., Santana M. J., Kline T. (2016). Multicenter Validation of the Quality of Trauma Care Patient-Reported Experience Measure (QTAC-PREM). *Journal of Trauma and Acute Care Surgery*.

[41] Bruyneel L., Tambuyzer E., Coeckelberghs . E. (2017). New Instrument to Measure Hospital Patient Experiences in Flanders. *International Journal of Environmental Research and Public Health*.

[42] Gibbons E., Hewitson P., Morley D., Jenkinson C., Fitzpatrick R. (2015). The Outcomes and Experiences Questionnaire: Development and Validation. *Patient Related Outcome Measures*.

[43] Jenkinson C., Coulter A., Bruster S., Richards N. (2002). The Coronary Heart Disease In-Patient Experience Questionnaire (I-PEQ (CHD)): Results from the Survey of National Health Service Patients. *Quality of Life Research*.

[44] Wilde L., Larsson G. (2002). Development of a Short Form of the Quality from the Patient’s Perspective (QPP) Questionnaire. *Journal of Clinical Nursing*.

[45] Oltedal S., Garratt A., Bjertnaes Ø, Bjørnsdottìr M., Freil M., Sachs M. (2007). The NORPEQ Patient Experiences Questionnaire: Data Quality, Internal Consistency and Validity Following a Norwegian Inpatient Survey. *Scandinavian Journal of Public Health*.

[46] Rothman A. A., Park H., Hays R. D., Edwards C., Dudley R. A. (2008). Can Additional Patient Experience Items Improve the Reliability of and Add New Domains to the CAHPS Hospital Survey?. *Health Services Research*.

[47] Sjetne I. S., Bjertnaes O. A., Olsen R. V., Iversen H. H., Bukholm G. (2011). The Generic Short Patient Experiences Questionnaire (GS-PEQ): Identification of Core Items from a Survey in Norway. *BMC Health Services Research*.

[48] Skudal K. E., Garratt A. M., Eriksson B., Leinonen T., Simonsen J., Bjertnaes O. A. (2012). The Nordic Patient Experiences Questionnaire (NORPEQ): Cross-National Comparison of Data Quality, Internal Consistency and Validity in Four Nordic Countries. *BMJ Open*.

[49] Sullivan P. J., Harris M. L., Doyle C., Bell D. (2013). Assessment of the Validity of the English National Health Service Adult In-Patient Survey for Use within Individual Specialties. *BMJ Quality and Safety*.

[50] Wang X., Chen J., Yang Y., BurströM B., BurströM K. (2021). Validation of the Patient-Reported Experience Measure for Care in Chinese Hospitals (PREM-CCH). *International Journal for Equity in Health*.

[51] Webster T. R., Mantopoulos J., Jackson E. (2011). A Brief Questionnaire for Assessing Patient Healthcare Experiences in Low-Income Settings. *International Journal for Quality in Health Care*.

[52] Wolf A., Olsson L.-E., Taft C., Swedberg K., Ekman I. (2012). Impacts of Patient Characteristics on Hospital Care Experience in 34,000 Swedish Patients. *BMC Nursing*.

[53] Wong E. L., Coulter A., Cheung A. W., Yam C. H., Yeoh E. K., Griffiths S. (2013). Validation of Inpatient Experience Questionnaire. *International Journal for Quality in Health Care*.

[54] Wong E. L. Y., Coulter A., Hewitson P. (2014). Patient Experience and Satisfaction With Inpatient Service: Development of Short Form Survey Instrument Measuring the Core Aspect of Inpatient Experience. *PLoS One*.

[55] Labarere J., Francois P., Auquier P., Robert C., Fourny M. (2001). Development of a French Inpatient Satisfaction Questionnaire. *International Journal for Quality in Health Care*.

[56] Iversen H. H., Haugum M., Bjertnaes O. (2022). Reliability and Validity of the Psychiatric Inpatient Patient Experience Questionnaire—Continuous Electronic Measurement (PIPEQ-CEM). *BMC Health Services Research*.

[57] Tobiano G., Marshall A. P., Gardiner T., Jenkinson K., Shapiro M., Ireland M. (2022). Development and Psychometric Testing of the Patient Participation in Bedside Handover Survey. *Health Expectations: An International Journal of Public Participation in Health Care & Health Policy*.

[58] Zhou W., Xie G., Yu Y., Gong H., Xiao S. (2022). Patients’ and Family Members’ Experiences of Psychiatric Inpatient Services in China: A Comparison Based on a Dyadic Design. *Social Psychiatry and Psychiatric Epidemiology*.

[59] Hansen C. F., Jensen J., Odgaard A. (2022). Four of Five Frequently Used Orthopedic PROMs Possess Inadequate Content Validity: A COSMIN Evaluation of the mHHS, HAGOS, IKDC-SKF, KOOS and KNEES-ACL. *Knee Surgery, Sports Traumatology, Arthroscopy*.

[60] Zegota S., Becker T., Hagmayer Y., Raupach T. (2022). Using Item Response Theory to Appraise Key Feature Examinations for Clinical Reasoning. *Medical Teacher*.

[61] Li H., Ding N., Zhang Y., Liu Y., Wen D. (2017). Assessing Medical Professionalism: A Systematic Review of Instruments and Their Measurement Properties. *PLoS One*.

[62] MuñOZ-GarcíA L. E., GóMEZ-Berrocal C., GuilléN-Riquelme A., Sierra J. C. (2023). Measurement Invariance across Sexual Orientation for Measures of Sexual Attitudes. *International Journal of Environmental Research and Public Health*.

[63] Lee J., Lee E. H., Chae D. (2021). eHealth Literacy Instruments: Systematic Review of Measurement Properties. *Journal of Medical Internet Research*.

[64] Manalili K., Santana M. J. (2021). Using Implementation Science to Inform the Integration of Electronic Patient-Reported Experience Measures (ePREMs) Into Healthcare Quality Improvement: Description of a Theory-Based Application in Primary Care. *Quality of Life Research*.

[65] Tian D., Hoehner C. M., Woeltje K. F., Luong L., Lane M. A. (2021). Disrupted and Restored Patient Experience With Transition to New Electronic Health Record System. *Journal of Patient Experience*.

[66] Bastemeijer C. M., Boosman H., Zandbelt L., Timman R., de Boer D., Hazelzet J. A. (2020). Patient Experience Monitor (PEM): The Development of New Short-form Picker Experience Questionnaires for Hospital Patients with a Wide Range of Literacy Levels. *Patient Related Outcome Measures*.

[67] van der Vleuten C. P. (1996). The Assessment of Professional Competence: Developments, Research and Practical Implications. *Advances in Health Sciences Education*.

