# Supplementary material for: Patient-Reported Experience Measures in Adult Inpatient Settings: A Systematic Review
Source: J Nurs Manag. 2024 Nov 23;2024:5166392. doi: 10.1155/jonm/5166392 (PMC11925316; doi:10.1155/jonm/5166392)
Supplement: Supporting Information — Additional supporting information can be found online in the Supporting Information section. [file 5166392.f1.docx]

# Search strategies

(#1 AND #2 AND #3 AND #4) NOT #5

#1:

patient experience*

patient's experience

inpatient experience

patients' medical experience*

patient reported experience

customer experience*

care experience*

hospital experience*

Patients' perceptions of care

#2:

inpatients[MeSH Terms]

inpatient*

hospitalization/hospitalisation

hospitalized/hospitalised

Patient Admission[MeSH Terms]

Patient Admit*

Patient Admission*

patient discharge[MeSH Terms]

patient discharge*

hospital discharge*

ward

"day patient"

Day Care, Medical[MeSH Terms]))

medical unit

"hospital stay"

#3:

Survey*

Questionnaire*

Instrument*

Inventory*

Measure*

Tool*

Scale*

#4:

Index

Indices

indicator*

clinimetr* or clinometr*

assessment

evaluat*

psychometric*

validat*

validity

Internal consistency

(reproducib* or reliab* or unreliab* or valid* or coefficient of variation or coefficient or homogeneity or homogeneous or internal consistency)

(cronbach* and (alpha or alphas))

(item and (correlation* or selection* or reduction*))

(test–retest or (test and retest))

(reliab* and (test or retest))

#5:

emergen*

outpatient

"nurse experience"

"doctor experience"

"physician experience"

# Search results

**Pubmed**

#1

(((((((patient experience*[Title/Abstract]) OR (patient's experience[Title/Abstract])) OR (inpatient experience[Title/Abstract])) OR (patients' medical experience*[Title/Abstract])) OR (patient reported experience[Title/Abstract])) OR (customer experience*[Title/Abstract])) OR (care experience*[Title/Abstract])) OR (hospital experience*[Title/Abstract])

33234

#2

((((((((((((((((inpatients[MeSH Terms]) OR (patient Admission[MeSH Terms])) OR (patient discharge[MeSH Terms])) OR (Day Care, Medical[MeSH Terms])) OR (inpatient*[Title/Abstract])) OR (hospitalization[Title/Abstract])) OR (hospitalisation[Title/Abstract])) OR (hospitalized[Title/Abstract])) OR (hospitalised[Title/Abstract])) OR (Patient Admit*[Title/Abstract])) OR (Patient Admission*[Title/Abstract])) OR (patient discharge*[Title/Abstract])) OR (hospital discharge*[Title/Abstract])) OR (ward[Title/Abstract])) OR ("day patient"[Title/Abstract])) OR (medical unit[Title/Abstract])) OR ("hospital stay"[Title/Abstract])

603,642

#3

((((emergen*[Title]) OR (outpatient[Title])) OR ("nurse experience"[Title])) OR ("doctor experience"[Title])) OR ("physician experience"[Title])

173,949

#4

(((((((((((((((((((((((((survey*[Title/Abstract]) OR (Questionnaire*[Title/Abstract])) OR (Surveys and Questionnaires[MeSH Terms])) OR (Instrument*[Title/Abstract])) OR (Inventory*[Title/Abstract])) OR (Measure*[Title/Abstract])) OR (Tool*[Title/Abstract])) OR (Scale*[Title/Abstract])) OR (Index[Title/Abstract])) OR (Indices[Title/Abstract])) OR (indicator*[Title/Abstract])) OR (clinimetr*[Title/Abstract])) OR (clinometr*[Title/Abstract])) OR (assessment[Title/Abstract])) OR (evaluat*[Title/Abstract])) OR (psychometric*[Title/Abstract])) OR (psychometrics[MeSH Terms])) OR (validat*[Title/Abstract])) OR (validity[Title/Abstract])) OR (Internal consistency[Title/Abstract])) OR (reproducibility of results[MeSH Terms])) OR ((reliab*[Title/Abstract] AND (test[Title/Abstract] OR retest[Title/Abstract])))) OR ((test–retest[Title/Abstract] OR (test[Title/Abstract] AND retest[Title/Abstract])))) OR ((item[Title/Abstract] AND (correlation*[Title/Abstract] OR selection*[Title/Abstract] OR reduction*[Title/Abstract])))) OR ((cronbach*[Title/Abstract] AND (alpha[Title/Abstract] OR alphas[Title/Abstract])))) OR ((reproducib*[Title/Abstract] OR reliab*[Title/Abstract] OR unreliab*[Title/Abstract] OR valid*[Title/Abstract] OR coefficient of variation[Title/Abstract] OR coefficient[Title/Abstract] OR homogeneity[Title/Abstract] OR homogeneous[Title/Abstract] OR internal consistency[Title/Abstract]))

11,302,516

#5: (#1 AND #2 AND #4) NOT #3: 2240

#6: #5 FLITERS:ENGLISH: **2178**

**Embase:**

1 ((patient* OR inpatient* OR care OR customer* OR medical OR hospital*) NEAR/2 experience*):ab,kw,ti 231030

2 (patient* NEAR/2 'perceptions of care'):ab,kw,ti 187

3 #1 or #2 231170

4 'hospital patient'/exp 235,610

5 'hospital admission'/exp 267,452

6 'hospital discharge'/exp 176,036

7 'day care'/exp 13950

8 inpatient*:ab,kw,ti 47644

9 hospitali*ation:ab,kw,ti 27421

10 hospitali*ed:ab,kw,ti 47183

11 ‘patient admit*’:ab,kw,ti 150

12 'patient admission':ab,kw,ti 143

13 'ward*':ab,kw,ti 19407

14 ((patient* OR hospital) NEAR/2 discharge*):ab,kw,ti 8926

15 (medical NEAR/2 unit):ab,kw,ti 1144

16 'day patient':ab,kw,ti 99

17 'hospital stay':ab,kw,ti 3773

18 4 or 5 or 6 or 7 or 8 or 9 or 10 or 11 or 12 or 13 or 14 or 15 or 16 or 17

697562

19 emergen*:ti 190025

20 outpatient*:ti 60718

21 ((nurse* OR doctor* OR physician*) NEAR/2 experience*):ti 3565

22 19 or 20 or 21 253,291

23 'survey'/exp OR 'questionnaire'/exp OR 'devices'/exp OR 'inventory'/exp OR 'instruments'/exp OR 'measurement'/exp OR 'tool'/exp OR 'scale'/exp OR 'index'/exp OR 'indicator'/exp OR 'clinimetrics'/exp OR 'assessment'/exp OR 'evaluation study'/exp OR 'psychometry'/exp OR 'validity'/exp OR 'reliability'/exp 8,299,941

24 measure* OR indice* OR psychometric* OR validat*:ab,ti,kw 6,258,883

25 'test retest' OR 'test and retest':ab,ti,kw 45,139

26 'item correlation*' OR 'item selection*' OR 'item reduction':ab,ti,kw 3248

27(reproducib* or reliab* or unreliab* or valid* or coefficient of variation or coefficient or homogeneity or homogeneous or internal consistency):ab,ti,kw 53426

28 23 or 24 or 25 or 26 or 27 12,330,867

29 #3 AND #18 AND #28 NOT #22 7028

39 #3 AND #18 AND #28 NOT #22 AND [english]/lim 6879

40#30 AND ('article'/it OR 'article in press'/it OR 'letter'/it OR 'preprint'/it OR 'review'/it OR 'short survey'/it) **3711**

**Cinahl**

#1 AB ( (patient* or inpatient* or care or custom or medical or hospital) N2 experience* ) OR AB patient* W2 'perceptions of care' OR TI patient* W2 'perceptions of care' OR TI ( (patient* or inpatient* or care or custom or medical or hospital) N2 experience* )

78,900

#2

AB ( inpatient* OR hospitali*ation OR hospitali*ed ) OR AB ( patient admission* OR patient admit* OR hospital discharge* OR 'day patient' OR ward OR 'hospital stay' ) OR AB ( (patient N2 discharge*) or (medical N2 unit) ) OR TI ( inpatient* OR hospitali*ation OR hospitali*ed ) OR TI ( patient admission* OR patient admit* OR hospital discharge* OR 'day patient' OR ward OR 'hospital stay' ) OR TI ( (patient N2 discharge*) or (medical N2 unit) ) OR MH inpatients OR MH patient admission OR MH day care OR MH patient discharge

164,906

#3

TI ( emegen* OR outpatient* ) OR TI ( (nurse* OR doctor* OR physician*) N1 experience* )

20,878

#4

MH surveys OR MH questionnaires OR MH scales OR MH evaluation OR MH psychometrics OR MH ( reliability and validity ) OR AB ( survey* OR questionnaire* OR instrument* OR inventor* OR measure* OR measurement* OR tool* OR scale* OR index OR indice* OR indicator* OR clinimet* OR clinometr* OR assessment* OR evaluat* OR psychometric* OR validity OR reliability ) OR TI ( survey* OR questionnaire* OR instrument* OR inventor* OR measure* OR measurement* OR tool* OR scale* OR index OR indice* OR indicato r* OR clinimet* OR clinometr* OR assessment* OR evaluat* OR psychometric* OR validity OR reliability) 2,657,153

#5 1 and 2 and 4 not 3 3184

#6 #5 Narrow by Language: english 3028

Import into endnote  **2975**

**PSYCINFO**

#S1

AB ( inpatient* or hospitali*ation or hospitali*ed ) OR AB ( Patient Admission* or Patient Admit* or hospital discharge* or "day patient" or ward or "hospital stay" ) OR AB ( patient n2 discharge* or medical n2 unit ) OR TI ( inpatient* or hospitali*ation or hospitali*ed ) OR TI ( Patient Admission* or Patient Admit* or hospital discharge* or "day patient" or ward or "hospital stay" ) OR TI ( patient n2 discharge* or medical n2 unit )

132,637

#S2

( AB ( inpatient* or hospitali*ation or hospitali*ed ) OR AB ( Patient Admission* or Patient Admit* or hospital discharge* or "day patient" or ward or "hospital stay" ) OR AB ( patient n2 discharge* or medical n2 unit ) OR TI ( inpatient* or hospitali*ation or hospitali*ed ) OR TI ( Patient Admission* or Patient Admit* or hospital discharge* or "day patient" or ward or "hospital stay" ) OR TI ( patient n2 discharge* or medical n2 unit ) ) AND ( AB (Patient* W2 perceptions of care) OR AB ( (patient* or inpatient* or care or customer or medical or hospital) N2 experience ) OR TI (Patient* W2 perceptions of care) OR TI ((patient* or inpatient* or care or customer or medical or hospital) N2 experience ) ) )

3,827

#S3

AB (Survey* OR Questionnaire* OR Instrument* OR Inventory* OR Measure* OR Tool* OR Scale* OR Index OR Indices OR indicator* OR clinimetr* OR clinometr* OR assessment OR evaluat* OR psychometric* OR validat* OR validity OR Internal consistency) OR TI(Survey* OR Questionnaire* OR Instrument* OR Inventory* OR Measure* OR Tool* OR Scale* OR Index OR Indices OR indicator* OR clinimetr* OR clinometr* OR assessment OR evaluat* OR psychometric* OR validat* OR validity OR Internal consistency) OR AB(reproducib* or reliab* or unreliab* or valid* or coefficient of variation or coefficient or homogeneity or homogeneous or internal consistency) OR TI(reproducib* or reliab* or unreliab* or valid* or coefficient of variation or coefficient or homogeneity or homogeneous or internal consistency) OR AB(cronbach* and (alpha or alphas)) OR TI(cronbach* and (alpha or alphas)) OR TI(item and (correlation* or selection* or reduction*)) OR AB(item and (correlation* or selection* or reduction*)) OR TI(test–retest or (test and retest)) OR AB(test–retest or (test and retest))

2,310,084

#S4

TI(emergen* or outpatient or (nurse* or doctor* or physician*) n1 experience)

29401

#S5

(S2 and S3) NOT S4

2013

Limiters - Language: English

**1902**

**PROSQUEST**

S1

mesh(inpatients) OR mesh(Patient admission) OR mesh(Day care) OR mesh(Patient discharge) OR mesh(hospitalization) OR ab(inpatient* OR hospitali*ation OR hospitali*ed OR Patient Admission* OR Patient Admit* OR hospital discharge* OR "day patient" OR ward OR "hospital stay") OR (ab(patient NEAR/2 discharge*) OR ab(medical NEAR/2 unit) OR ti(inpatient* OR hospitali*ation OR hospitali*ed OR Patient Admission* OR Patient Admit* OR hospital discharge* OR "day patient" OR "in hospital" OR "hospital stay") OR ti(patient NEAR/2 discharge*) OR ti(medical NEAR/2 unit))

all 349,108

S2

abstract(Patient* Pre/2 perceptions of care) OR abstract((Patient* OR inpatient* OR care OR customer OR medical OR hospital) NEAR/2 experience) OR title(Patient* Pre/2 perceptions of care) OR title((Patient* OR inpatient* OR care OR customer OR medical OR hospital) NEAR/2 experience)

All 49543

S3

(ti(emergen*) OR ti(outpatient) OR ti(nurse* n/1 experience) OR ti(doctor n/1 experience)) OR ti(physician* n/1 experience)

all 92961

S4

abstract(Survey* OR Questionnaire* OR Instrument* OR Inventory* OR Measure* OR Tool* OR Scale* OR Index OR Indices OR indicator* OR clinimetr* OR clinometr* OR assessment OR evaluat* OR psychometric* OR validat* OR validity OR Internal consistency) OR title(Survey* OR Questionnaire* OR Instrument* OR Inventory* OR Measure* OR Tool* OR Scale* OR Index OR Indices OR indicator* OR clinimetr* OR clinometr* OR assessment OR evaluat* OR psychometric* OR validat* OR validity OR Internal consistency) OR abstract(reproducib* or reliab* or unreliab* or valid* or coefficient of variation or coefficient or homogeneity or homogeneous or internal consistency) OR title(reproducib* or reliab* or unreliab* or valid* or coefficient of variation or coefficient or homogeneity or homogeneous or internal consistency) OR abstract(cronbach* and (alpha or alphas)) OR title(cronbach* and (alpha or alphas)) OR title(item and (correlation* or selection* or reduction*)) OR abstract(item and (correlation* or selection* or reduction*)) OR title(test–retest or (test and retest)) OR abstract(test–retest or (test and retest))

All 3779054

((mesh(inpatients) OR mesh(Patient admission) OR mesh(Day care) OR mesh(Patient discharge) OR mesh(hospitalization) OR ab(inpatient* OR hospitali*ation OR hospitali*ed OR Patient Admission* OR Patient Admit* OR hospital discharge* OR "day patient" OR ward OR "hospital stay") OR (ab(patient NEAR/2 discharge*) OR ab(medical NEAR/2 unit) OR ti(inpatient* OR hospitali*ation OR hospitali*ed OR Patient Admission* OR Patient Admit* OR hospital discharge* OR "day patient" OR "in hospital" OR "hospital stay") OR ti(patient NEAR/2 discharge*) OR ti(medical NEAR/2 unit))) AND (abstract(Patient* Pre/2 perceptions of care) OR abstract((Patient* OR inpatient* OR care OR customer OR medical OR hospital) NEAR/2 experience) OR title(Patient* Pre/2 perceptions of care) OR title((Patient* OR inpatient* OR care OR customer OR medical OR hospital) NEAR/2 experience)) AND (abstract(Survey* OR Questionnaire* OR Instrument* OR Inventory* OR Measure* OR Tool* OR Scale* OR Index OR Indices OR indicator* OR clinimetr* OR clinometr* OR assessment OR evaluat* OR psychometric* OR validat* OR validity OR Internal consistency) OR title(Survey* OR Questionnaire* OR Instrument* OR Inventory* OR Measure* OR Tool* OR Scale* OR Index OR Indices OR indicator* OR clinimetr* OR clinometr* OR assessment OR evaluat* OR psychometric* OR validat* OR validity OR Internal consistency) OR abstract(reproducib* or reliab* or unreliab* or valid* or coefficient of variation or coefficient or homogeneity or homogeneous or internal consistency) OR title(reproducib* or reliab* or unreliab* or valid* or coefficient of variation or coefficient or homogeneity or homogeneous or internal consistency) OR abstract(cronbach* and (alpha or alphas)) OR title(cronbach* and (alpha or alphas)) OR title(item and (correlation* or selection* or reduction*)) OR abstract(item and (correlation* or selection* or reduction*)) OR title(test–retest or (test and retest)) OR abstract(test–retest or (test and retest)))) NOT (ti(emergen*) OR ti(outpatient) OR ti(nurse* n/1 experience) OR ti(doctor n/1 experience) OR ti(physician* n/1 experience))

(S1 and S2 and S4) not S3

4,075

Filters：English，journals，report，thesis

**3,916**

**Cochrane**

#1 (inpatient* or hospitali*ation or hospitali*ed or Patient Admission* or Patient Admit* or hospital discharge* or "day patient" or ward or "hospital stay"):ti,ab,kw OR (patient NEAR/2 discharge*):ti,ab,kw OR (medical NEAR/2 unit):ti,ab,kw (Word variations have been searched) with Cochrane Library publication date Between Jan 1900 and Mar 2023 152135

#2 MeSH descriptor: [Patient Admission] explode all trees 823

#3 MeSH descriptor: [Inpatients] explode all trees 1641

#4 MeSH descriptor: [Day Care, Medical] explode all trees 305

#5 MeSH descriptor: [Patient Discharge] explode all trees 2675

#6 #1 OR #2 OR #3 OR #4 OR #5 with Cochrane Library publication date Between Jan 1900 and Mar 2023 152278

#7 (Patient* NEAR/2 perceptions of care):ti,ab,kw OR ((Patient* or inpatient* or care or customer or medical or hospital) NEAR/2 experience):ti,ab,kw with Cochrane Library publication date Between Jan 1900 and Mar 2023 7678

#8 (Survey* OR Questionnaire* OR Instrument* OR Inventory* OR Measure* OR Tool* OR Scale* OR Index OR Indices OR indicator* OR clinimetr* OR clinometr* OR assessment OR evaluat* OR psychometric* OR validat* OR validity OR Internal consistency):ti,ab,kw OR (reproducib* or reliab* or unreliab* or valid* or coefficient of variation or coefficient or homogeneity or homogeneous or internal consistency):ti,ab,kw OR (cronbach* NEAR/2 alpha*):ti,ab,kw OR (item NEAR/2 (correlation* or selection* or reduction*)):ti,ab,kw OR (test–retest):ti,ab,kw (Word variations have been searched) with Cochrane Library publication date Between Jan 1900 and Mar 2023 1345240

#9 #6 AND #7 AND #8 1996

#10 (emergen* or outpatient or (nurse* or doctor* or physician*) near/1 experience):ti (Word variations have been searched) 24427

#11 #9 NOT #10  **1732**
